# Zika epidemic and social inequalities: Brazil and its fate: Epidemia de zika e desigualdades sociais: Brasil e seu destino

**DOI:** 10.1590/1516-3180.2016.13421602

**Published:** 2015-03-17

**Authors:** Paulo Andrade Lotufo

**Affiliations:** 1 MD, DrPH. Full Professor, Department of Internal Medicine, Faculdade de Medicina da Universidade de São Paulo (FMUSP), São Paulo, SP, Brazil.

In January 2001, I wrote an editorial on this page with the title "Coffee, Samba, Football, AIDS and Social Inequality in Brazil." The first paragraph described a contradiction within the Brazilian National Health System: "The most important newspapers and magazines worldwide (The New York Times, Le Monde, The Wall Street Journal, Time Magazine) have been publishing the tremendous results from the Brazilian AIDS program. This is a combination of preventive measures and free distribution of antiretroviral drugs with an impressive fall in hospitalizations and mortality. Now, the Brazilian program has become the paramount public health effort for halting the AIDS epidemic to be followed in other places, especially the sub-Saharan African countries. In contrast, the Health Department of São Paulo State is warning about increasing case-fatality rates of tuberculosis. How can this contradiction be explained for a non-Brazilian?"^1^

Imagine if I published the same editorial again, just changing the phrase "about increasing case-fatality rates of tuberculosis" by "the recent incidence of Zika virus infection and new cases of microcephaly". Readers might think that they could detect an instance of self-plagiarism. Unfortunately, this is not a lazy way to write a comment by copying an older one. The fact is that the players can change, but the play is the same on the stage of Brazilian public health.

The question transcribed above was answered in that editorial in 2001: "for us, Brazilian physicians and medical researchers, the answer is very easy. AIDS is a disease that has afflicted people with real prestige in Brazilian circles of power. In summary: journalists, popular music stars, soap-opera actors and actresses, physicians, scientists, military officers, priests and other well-born citizens who traveled to the USA during the early 1980s and were infected by sexual intercourse in New York City and San Francisco. After that, they spread the virus and disease among Brazilians who had never dreamed of going up the steps on an airplane. Until the early 1990s, in São Paulo City, the incidence of AIDS cases continued to be greater among affluent people than among poor ones. As the spread of AIDS became a menace to the abovementioned professions, it was easy to pressure the Ministry of Health, State Departments of Health, and other authorities to increase the budget for AIDS control and treatment, including sometimes a shift of resources from other programs".^1^

For more than a decade, the Brazilian AIDS program was self-proclaimed as "an example for the world". In contrast, the control over dengue has deteriorated year by year. Despite the manifest failure of control over the dengue epidemic, which has been conceded by most of the communicable disease epidemiologists in Brazil, in an article in *The Lancet*, the federal budget for AIDS has been increasing and the budget for controlling vectors like *Aedes aegypti* is still declining.^2^

## THE CASE OF PRE-EXPOSURE PROPHYLAXIS FOR HIV CONTROL

One year ago, the voracious appetite for more money propelled the Brazilian authorities, physicians and AIDS activists to stay on the front-line of the fight against AIDS by advocating adoption by the Brazilian National Health System of a strategy called "pre-exposure prophylaxis" for HIV control. This is a very controversial proposal that will cost $ 13,000/year/per capita. I was invited to wrote an op-ed article in the most-read Brazilian newspaper^3^ rebutting the proposal made in the same issue by Luiz Loures, the Deputy Executive Director of UNAIDS (the United Nations program for combating AIDS), who stated that "PreP should be the opportunity to finish up the AIDS epidemic." (sic)^4^

The basis of my reasoning was that scientific articles supporting the use of pre-exposure prophylaxis presented several limitations, not only from a strictly scientific view, but also from a strategic point of view. In my view, this proposal was a step backwards in relation to preventive efforts towards halting both AIDS and also other sexually transmitted diseases.

## THE MEDICAL-INDUSTRIAL COMPLEX

My reasoning in that article in *Folha de S.Paulo* was to refer to Arnold Relman (former editor of the *New England Journal of Medicine* ), who wrote a seminal editorial about the concept of the medical-industrial complex. He described this situation as similar to the one of the "military-industrial complex" that Dwight Eisenhower described during his US Presidential term in the 1950s.

In fact, we are under heavy pressure from the "medical- industrial complex," now using UNAIDS as its spokesperson, to take money from other activities such as vector control, in order to put it into the pockets of the AIDS medical industrial complex. What is incredible in this story is that the proposal for pre-exposure prophylaxis relates only to one medicine: Truvada, manufactured by Gilead, in California. The leftist watchdogs who start barking if there is even a one-day delay in free distribution of any HIV medication did not even whimper about any conflict of interest in this case.

One year after this debate, the same newspaper, Folha de S. Paulo published an article signed by the "Vice Brazil Organization" regretting that the Brazilian National Health System still did not include pre-exposure prophylaxis as free medicine. Furthermore, the author stimulated readers to start lawsuits against the Brazilian National Health System to get free drugs so that people could indulge in unsafe sexual intercourse.^5^

## THE SAME PLAYERS ON THE STAGE

At the same time that pre-exposure prophylaxis was becoming a motive for great concern within the media located in São Paulo and Rio de Janeiro, less fortunate people in the poorest areas of the country were suffering from dengue, chikungunya and Zika. To the best of my knowledge, no one involved in AIDS activism is either creating some networks for supporting parents and children afflicted by the consequences of Zika infection, or is demanding more funds for combating zika infection.

An editorial by Richard Horton in The Lancet took the view that the Zika epidemic is an opportunity for Brazil to change its public health system.^6^ I am skeptical. The reason why the Zika epidemic exists is that the dengue epidemic was neglected because it was limited to poor people, just like tuberculosis was neglected 20 years ago. The President of Brazil, Ms. Dilma Roussef, is mobilizing her staff to halt the *Aedes aegypti* outbreak using military metaphors. She blamed her predecessors, despite the fact that this is her second term. It would be better for us if the President, the Brazilian Congress, the Ministry of Health, the media, the medical establishment and leftist activists could only acknowledge that we have been doing things wrongly for a long time now. Zika is only the play of the season of social inequalities in Brazil.
